# High-resolution ultrasound with Doppler as a confirmatory diagnostic method in retronychia^⋆^

**DOI:** 10.1016/j.abd.2021.07.013

**Published:** 2024-01-29

**Authors:** Cristina Vélez Arroyave, Laura Carvajal Betancur, Ángela María Londoño García, Leonard Pacheco Peñaranda

**Affiliations:** a Department of Dermatology, Universidad CES, Medellín, Colombia; b Department of Dermatology, Epidemiologist, Universidad CES, Medellín, Colombia; c Department of Radiology, Expert in Dermatological Ultrasound, Responsible for Sonoderma, Medellin, Colombia

Dear Editor,

We present the case of a 45-year-old female patient who was referred to the dermatologist with erythema, pain, and discharge in the left hallux. This condition had been present for four months and was resistant to topical and oral antibiotics. She had active secretion and complete loss of the union of the proximal fold in the affected nail, henceforth chronic paronychia was the original diagnosis (Fig. 1). A high-resolution ultrasound with Doppler analysis of the nail apparatus was requested. The findings established the diagnosis of rethonychia (Figs. 2‒4). Then we decided to perform a surgical intervention.

**Figure 1:**
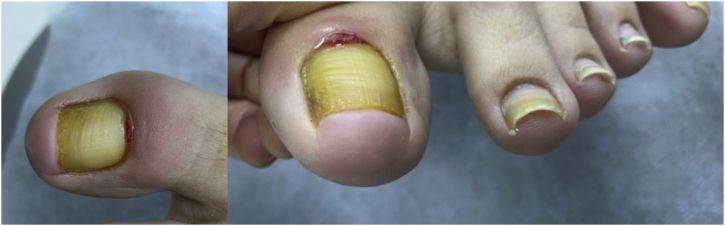
There is absence of plate growth due to complete loss of the union of the proximal fold.

**Figure 2:**
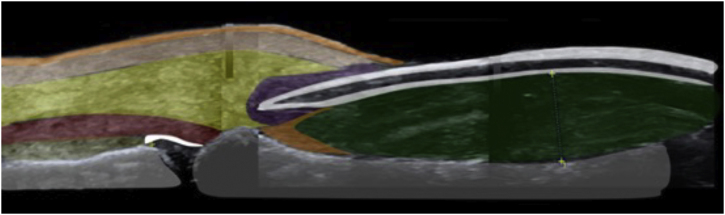
Sonoanatomy of the nail apparatus in retronychia: nail plate (white), nail bed (green), nail matrix (orange), inflammatory halo (purple), extensor tendon (red), subcutaneous tissue (yellow), dermis (pink), epidermis (orange) and phalanx (gray).

**Figure 3:**
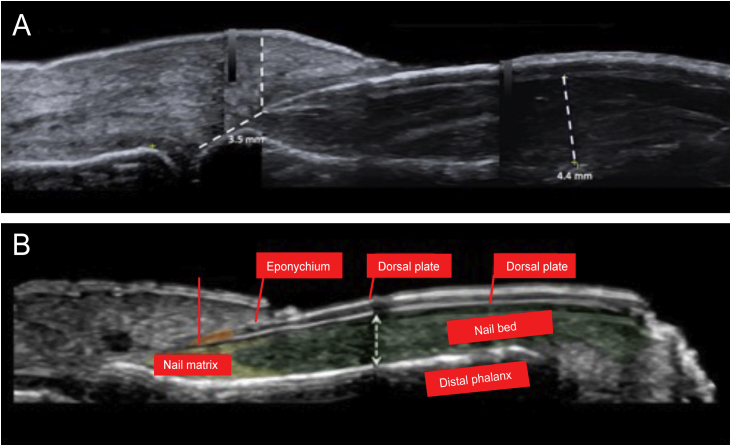
(a) High-resolution skin ultrasound with Doppler analysis performed with a 22 Mhz translator showed thickening of the nail bed with hypoechoic appearance. It measured 4.4 mm and did not show any signs of hypervascularization on the Doppler exam. However, it did show a reduction in the space between the origin of the nail plate and the base of the distal phalanx: its measure was approximately 3.5 mm and presented a hypoechoic inflammatory halo. (b) Normal anatomy of the nail.

**Figure 4:**
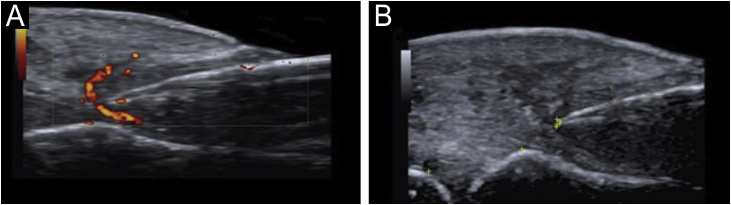
(a) Power Doppler around the nail plate. (b) Visualization of thickening of the nail fold (dermis and hypodermis) of 5 mm.

Retronychia is a disorder of the nail apparatus in which there is an abnormal growth of the nail plate within the proximal fold, leading to the formation of several generations of misaligned nail plates under the fold.^1^, ^2^

Retronychia affects middle-aged adults, mostly females.^3^ It is characterized by unilateral involvement and affects almost exclusively the hallux.^4^ Repeated trauma, pregnancy, puerperium, use of tight footwear, and anatomical alterations such as fingers in claws or curved nails have been described as associated factors.^4^

After persistent minor traumas, the process begins with an interruption of the growth of the nail plate that leads to its misalignment and incomplete separation from the matrix, where the nail plate loses its fixation in the proximal part of the bed but remains attached to the matrix in the lateral aspects.^5^ The nail plate moves in a retrograde direction and becomes embedded in the proximal nail fold, leading to inflammation and large tissue formation.^1^ The new nail plate will push the old one upwards, leading to abnormal growth and inflammation, thus becoming a vicious cycle, where up to four generations of nail plates can be superimposed under the proximal nail fold. Retronychia often manifests as a chronic paronychia that is resistant to antimicrobial therapy.^6^

The diagnosis of retronychia is based primarily on clinical aspects and is confirmed by imaging methods. There are different imaging modalities to study the ungular apparatus, including simple radiography, ultrasound, and Magnetic Resonance Imaging (MRI).^7^ Ultrasound has proven to be useful; it has the advantage that it is a non-invasive method, it is accessible and helps in surgical planning.^8^ The type of ultrasound used for the diagnosis in the case presented is a high-resolution method with Doppler analysis, which is different from classical ultrasound. It uses transducers with higher frequencies, up to 22 MHz, that provide a complete and real-time view of the ungular apparatus. It differs by the addition of Doppler analysis and requires a trained person. In the literature, there is evidence of the use of this type of ultrasound to evaluate nail diseases. High-resolution ultrasound proves to be a precise and complete tool where various characteristics of the ungual apparatus can be evaluated that leads to a high histological correlation of different nail conditions.^9^

MRI is also another option. It is excellent for the evaluation of tumors, especially vascular tumors. However, it is highly costly, and it also requires complete immobility of the affected limb, making it difficult to use in the pediatric population.^7^

Ultrasound criteria for rethonychia have been described:^5^

1. Presence of a hypoechoic halo surrounding the origin of the nail plate.
2. Distance (≤ v5.1 mm) between the origin of the nail plate and the base of the distal phalanx (thumbs and first toes) or a difference in this distance ≥ 0.5 mm compared to the contralateral healthy finger.
3. Proximal nail fold thickness ≥ 2.2 mm in men or ≥ 1.9 mm in women and/or a thickness ≥ 0.3 mm compared to the contralateral healthy finger.

If it is unilateral, it must meet these three criteria; if it is bilateral, it can meet any of the three criteria.

Other findings on ultrasound in retronychia are:^1^

• Two or more overlapping nail plates.
• Increased blood flow in the dermis of the proximal fold and the nail bed.

Possible causes of chronic paronychia such as infections, neoplasms, systemic diseases, and medications should be ruled out.^1^

The mainstay of treatment is based on surgical avulsion, performing a proximal approach to the old nail plate and the possible underlying ingrown plates.^1^ High-potency topical steroids could be used in conjunction with a bandage to fix the nail to the bed.^3^

To prevent retronychia it is important to recommend patients to wear comfortable shoes, avoid repetitive trauma, and in case of foot deformities these should be corrected.^2^

## Financial support

None declared.

## Authors’ contribution

Cristina Vélez Arroyave: Critical literature review; preparation and writing of the manuscript and study conception and planning.

Laura Carvajal Betancur: Critical literature review; preparation and writing of the manuscript and study conception and planning.

Ángela María Londoño García: Approval of the final version of the manuscript; effective participation in research orientation and study conception and planning.

Leonard Pacheco Peñaranda: Approval of the final version of the manuscript; effective participation in research orientation and study conception and planning.

## Conflicts of interest

None declared.
